# Quantified Coexpression Analysis of Central Amygdala Subpopulations

**DOI:** 10.1523/ENEURO.0010-18.2018

**Published:** 2018-02-06

**Authors:** Kenneth M. McCullough, Filomene G. Morrison, Jakob Hartmann, William A. Carlezon, Kerry J. Ressler

**Affiliations:** 1 Division of Depression and Anxiety Disorders, Department of Psychiatry, McLean Hospital, Harvard Medical School, Belmont, MA, 02478; 2 Behavioral Science Division, National Center for PTSD:Boston, MA, 02118; 3 VA Boston Healthcare System, Boston, MA; 4 Department of Psychiatry, Boston University School of Medicine, Boston, MA

**Keywords:** Amygdale, Coexpression, Crf, Sst, Prkcd, Tac2, Nts, fear, Somatostatin

## Abstract

Molecular identification and characterization of fear controlling circuitries is a promising path towards developing targeted treatments of fear-related disorders. Three-color *in situ* hybridization analysis was used to determine whether somatostatin (SOM, *Sst*), neurotensin (NTS, *Nts*), corticotropin-releasing factor (CRF, *Crf*), tachykinin 2 (TAC2, *Tac2*), protein kinase c-δ (PKC-δ, *Prkcd*), and dopamine receptor 2 (DRD2, *Drd2*) mRNA colocalize in male mouse amygdala neurons. Expression and colocalization was examined across capsular (CeC), lateral (CeL), and medial (CeM) compartments of the central amygdala. The greatest expression of *Prkcd* and *Drd2* were found in CeC and CeL. *Crf* was expressed primarily in CeL, while *Sst*-, *Nts*-, and *Tac2*-expressing neurons were distributed between CeL and CeM. High levels of colocalization were identified between *Sst*, *Nts*, *Crf*, and *Tac2* within the CeL, while little colocalization was detected between any mRNAs within the CeM. These findings provide a more detailed understanding of the molecular mechanisms that regulate the development and maintenance of fear and anxiety behaviors.

## Significance Statement

Functional and behavioral analysis of central amygdala microcircuits has yielded significant insights into the role of this nucleus in fear and anxiety related behaviors. However, precise molecular and locational description of examined populations is lacking. This publication provides a quantified regionally precise description of the expression and coexpression of six frequently examined central amygdala population markers. Most revealing, within the most commonly examined region, the posterior central lateral amygdala (CeL), four of these markers are extensively coexpressed, suggesting the potential for experimental redundancy. These data clarify circuit interaction and function and will increase relevance and precision of future cell type-specific reports.

## Introduction

The amygdala comprises a wide array of molecularly, electrophysiologically, and functionally distinct cell populations (Cassell et al., 1999; Ciocchi et al., 2010; Yu et al., 2016). Recent evidence suggests that distinct subpopulations play differential roles in fear and extinction learning (Herry et al., 2008; Haubensak et al., 2010; Li et al., 2013a; Kim et al., 2017). The characterization of molecularly identifiable neuronal populations is an important early step in developing improved treatments for fear and anxiety related disorders (McCullough et al., 2016).

Previous work has shown that the central lateral amygdala (CeL) contains a mutually inhibitory circuit that gates fear expression via the inhibition of central medial amygdala (CeM) output neurons (Herry et al., 2008; Ehrlich et al., 2009; Ciocchi et al., 2010; Letzkus et al., 2015). The CeL is often conflated with the central capsular division of the amygdala (CeC) although these regions have distinct projection patterns and potentially different roles in fear and anxiety; in the present manuscript we discuss these two regions separately (Jolkkonen and Pitkänen, 1998; Bourgeais et al., 2001).

The protein kinase c-δ (PKC-δ, *Prkcd*)-expressing neuron population has previously been shown to directly inhibit CeM output neurons, reducing activity in response to conditioned stimuli (CS) following fear conditioning and thus playing an important role in fear extinction learning (Herry et al., 2008; Ciocchi et al., 2010; Haubensak et al., 2010; Cai et al., 2014). The somatostatin (SOM, *Sst*) expressing population appears to be a counterpart of the PKC-δ population; activity of SOM neurons increases in response to CS following fear conditioning and activity in this population is both necessary and sufficient for the production of fear and defensive behaviors (Li et al., 2013b; Penzo et al., 2014; Yu et al., 2016). The tachykinin 2 (TAC2, *Tac2*) population plays a complementary role to the SOM population; activity of the *Tac 2* expressing population is both necessary and sufficient for fear learning (Andero et al., 2014; Andero et al., 2016).

In addition to SOM and TAC2, other neuropeptides have been implicated as playing critical roles in fear circuitry. In particular corticotropin-releasing factor (CRF, *Crf*)- and neurotensin (NTS, *Nts*)-expressing neurons are expressed in populations ideally situated and connected to participate in the central amygdala fear controlling circuit (Petrovich and Swanson, 1997). Both NTS and CRF have been shown to play important roles in fear learning and expression (Merali et al., 1998; Thompson et al., 2004; Yamauchi et al., 2007; Shilling and Feifel, 2008; Gafford and Ressler, 2015).

Dopamine plays a critical role in fear and extinction learning. Specifically, the differential distributions of dopamine receptors may have important implications for mediating fear behaviors (de la Mora et al., 2010; Abraham et al., 2014; Kwon et al., 2015). The dopamine receptor 2 (DRD2, *Drd2*) has been suggested to label a large population of neurons implicated in the development and maintenance of fear behaviors (Perez de la Mora et al., 2012; Kim et al., 2017).

Considering the large numbers of CeA cell populations that play parallel or complementary roles in fear behaviors, it is important to determine the extent to which these populations overlap. While much work has been completed identifying markers for behaviorally relevant neuronal populations, less has been done to examine the extent to which each of these populations is distinguishable on the basis of gene expression patterns. In the present investigation three-color *in situ* hybridization was used to determine the extent of overlap in expression of *Prkcd*, *Sst*, *Nts*, *Tac2*, *Crf*, and *Drd2*. Importantly, significant differences are found in distribution and overlap across the anterior-posterior (A-P) axis of the CeA, thus results are provided as both compressed across the CeA and split into anterior (A-P -0.8 to -1.2) and posterior (A-P -1.3 to -1.8) fractions (McDonald, 2003). Results suggest that within the CeC, *Prkcd* and *Drd2* label large nonoverlapping populations. Within the posterior CeL, *Sst*, *Tac2*, *Nts*, and *Crf* populations largely overlap. Of these *Sst* labels the largest population that contains the others markers to varying extents. Within the CeL, the *Prkcd* and *Drd2* populations largely do not overlap with each other or the other populations examined. The CeM has moderately sized *Sst*, *Tac2*, *Nts*, and *Crf* populations, but is largely devoid of *Prkcd*- and *Drd2*-labeled cells. Notably, unlike within the CeL, within the CeM, the *Sst*, *Tac2*, *Nts*, and *Crf* populations largely do not overlap suggesting important differences in the functional populations labeled by these markers in the CeL and CeM.

## Materials and Methods

### Animals

C57BL/6J mice were obtained from The Jackson Laboratory. All ten male mice were adult (10 weeks) at the time of tissue collection. All mice were group housed and maintained on a 12/12 h light/dark cycle. Mice were housed in a temperature-controlled colony and given unrestricted access to food and water. All procedures conformed to National Institutes of Health guidelines and were approved by McLean Hospital Institutional Animal Care and use Committee. All animals were killed, and tissue was collected together during light cycle at zeitgeber 3:00 P.M.

### RNA scope staining

Staining for mRNA of interest was accomplished using RNA Scope Fluorescent Multiplex 2.5 labeling kit. Probes used for staining are: mm-Nts-C1, mmNts-C2, mm-Tac2-C1, mm-Tac2-C2, mm-Sst-C1, mm-Sst-C2, mm-Crh-C1, mm,Prkcd-C1, mm-Prkcd-C3, mmDrd2-C3. Brains were extracted and snap-frozen in methyl-butane on dry ice. Sections were taken at a width of 16 µm. Staining procedure was completed to manufacturers specifications.

### Image acquisition

Images were acquired with experimenter blinded to probes used. Sixteen-bit images of each section were acquired on a Leica SP8 confocal microscope using a 10× objective. Within a sample images were acquired with identical settings for laser power, detector gain, and amplifier offset. Images were acquired as a z-stack of 10 steps of 0.5 µm each. Max intensity projections were then created and analyzed.

### Data analysis

The expression and coexpression of mRNA of different markers of interest was quantified in three areas CeC, CeL, and CeM. Images (approximate area) of regions were taken bilaterally from a minimum of one section from each of four animals for each marker pair (*n* > 8 amygdala/marker pair). Individual cells were identified based on DAPI staining of the nucleus. Cells were determined to be expressing marker when more than five fluorescent dots or an area of staining sufficient to contain five dots was clearly associated with a single nucleus. The width of a cell was considered to be twice the diameter of the nucleus. The distribution of cells across CeA nuclei was determined by dividing the number of labeled cells in a nucleus by the total number of labeled cells across all nuclei. The percentage of cells in a nucleus expressing a certain mRNA was determined by dividing the number of positive cells in a nucleus by the total number of DAPI-labeled nuclei in the nucleus. Experimenters blinded to the identity of probes completed all counts.

### Statistical analysis

Determination of the percentage of cells within a subcompartment expressing marker of interest was accomplished by dividing the total number of cells expressing the marker by the total number of DAPI-positive nuclei in the area and multiplying by 100. Determination of the percentage of a labeled population found in a certain subcompartment was accomplished by dividing the number of labeled cells in a compartment by the total number of labeled cells found in all compartments and multiplying by 100. Statistical analysis of the whether a labeled population was significantly different from the coexpressing component of that population was performed using the nonparametric Mann–Whitney test with GraphPad Prism software package.

## Results

Patterns of mRNA expression observed with *in situ* staining for colocalization of three marker experiments were identical to those observed from single labeling of each marker. Staining patterns were consistent with those observed in the literature and with those produced by the Allen Brain Institute. All six probes produced strong staining in the central CeA.

Each marker was examined individually to characterize its distribution across subcompartments of the CeA. Determination of subcompartment location was accomplished through examination of DAPI staining patterns. As the most popular available brain atlases (Paxinos and Allen Institute) differ somewhat on the locations of CeA subcompartments across the A-P axis of the amygdala the reference atlas provided through the Allen Brain Institute was used as a primary guide (Lein et al., 2007).

### Distribution of labeled cells

**Figure 1.**
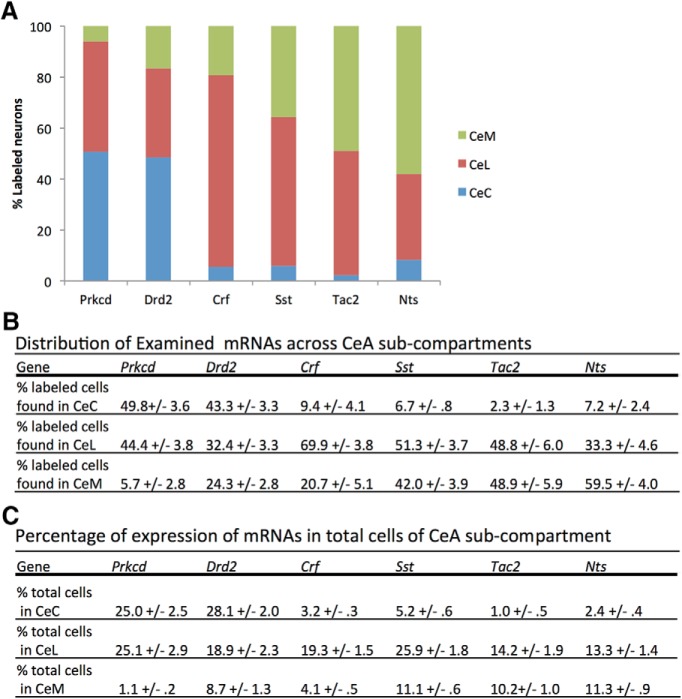
Distribution of examined mRNAs across CeA subcompartments. ***A***, Graphical representation of labeled cell distribution across CeA subcompartments. ***B***, Tabular results of data represented in ***A***. Each column presents distribution of all counted labeled cells in each CeA subcompartment ± SEM. ***C***, Labeled cells as percentage of total cells in subcompartment. Labeled cell counts presented as a percentage of total DAPI-positive nuclei examined within a nucleus ± SEM.

The distribution of total cells expressing mRNAs of interest was examined across CeA subcompartments (Fig. 1*A*,*B*) *Prkcd* staining was almost entirely contained within the CeC (49.8 ± 3.6% of labeled cells) and CeL (44.4 ± 3.8%) with only minority populations found within the CeM (5.7 ± 2.8%). *Drd2* was strongly expressed within the CeC (43.3 ± 3.3%) and CeL (32.4 ± 3.3%) with a smaller population within the CeM (24.3 ± 2.8%). *Crf* was primarily expressed within CeL (69.9 ± 3.8) with smaller populations in CeM (20.7 ± 5.1%) and CeC (9.4 ± 4.1%). *Sst*, *Tac2*, and *Nts* populations are primarily found in CeL (51.3 ± 3.7%, 48.8 ± 6.0%, and 33.3 ± 4.6%, respectively) and CeM (42.0 ± 5.9%, 48.9 ± 5.9%, and 59.5 ± 4.0%, respectively) with only small numbers of cells labeled in CeC (6.7 ± 0.8%, 2.3 ± 1.3%, and 7.2 ± 2.4%, respectively).

### Prevalence of labeled cells

Single labeling by marker mRNAs was examined to determine their prevalence within a subcompartment (Fig. 1*C*). This was completed by determining the proportion of labeled cells to the total number of DAPI-positive cells within a compartment. *Drd2* and *Prkcd* label large proportions of cells within the CeC (28.1 ± 2.0% of total DAPI-positive nuclei and 25.0 ± 2.5%, respectively) while other markers *Sst*, *Tac2*, *Nts*, and *Crf* label minority populations (5.2 ± 0.6%, 1.0 ± 0.5%, 2.4 ± 0.4%, 3.2 ± 0.3%, respectively). *Prkcd*, *Drd2*, *Sst*, *Tac2*, *Nts*, and *Crf* each label significant populations within the CeL (25.1 ± 2.9%, 18.9 ± 2.3%, 25.9 ± 1.8%, 14.2 ± 1.9%, 13.3 ± 1.4%, and 19.3 ± 1.5%, respectively). *Sst*, *Tac2*, and *Nts* label moderate populations within the CeM (11.1 ± 0.6%, 10.2 ± 1.0%, and 11.3 ± 0.9%, respectively), while *Prkcd*, *Drd*, and *Crf* label smaller proportions of cells (1.1 ± 0.2%, 8.7 ± 1.3%, and 4.1 ± 0.5%, respectively).

### Colocalization of CeA markers

**Figure 2.**
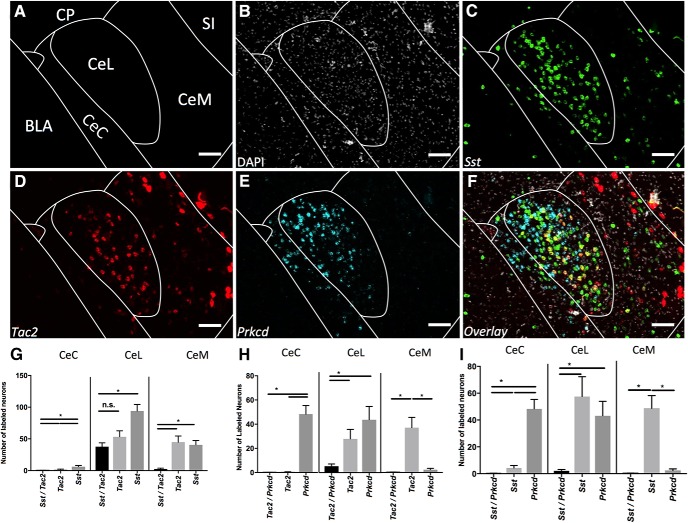
Coexpression of *Sst*, *Tac2*, and *Prkcd* (A-P -1.5). ***A***, Map of area examined. ***B***, DAPI stain (gray) of area examined. ***C***, *Sst* expression (green) is found strongly in the CeL and CeM. ***D***, *Tac2* expression (red) is found strongly in the CeL and CeM. ***E***, *Prkcd* (cyan) expression is found strongly in the CeC and CeL. ***F***, Overlay of ***B–E*** reveals strong overlap in expression of *Sst* and *Tac2* in CeL but not CeM. *Prkcd* does not highly coexpress in any area. Scale bar: 50 µm. ***G***, Quantification of single expressing cells and coexpressing *Sst* and *Tac2* cells in CeC, CeL, and CeM. Bars represent the mean number of (co)expressing cells in each subcompartment. ***H***, Quantification of single expressing cells and coexpressing *Tac2* and *Prkcd* cells in CeC, CeL, and CeM. Bars represent the mean number of (co)expressing cells in each subcompartment. ***I***, Quantification of single expressing cells and coexpressing *Sst* and *Prkcd* cells in CeC, CeL, and CeM. Bars represent the mean number of (co)expressing cells in each subcompartment. Data presented as mean ± SEM where *p < 0.05 difference between single and double-labeled populations (Mann–Whitney *U* test).

**Figure 3.**
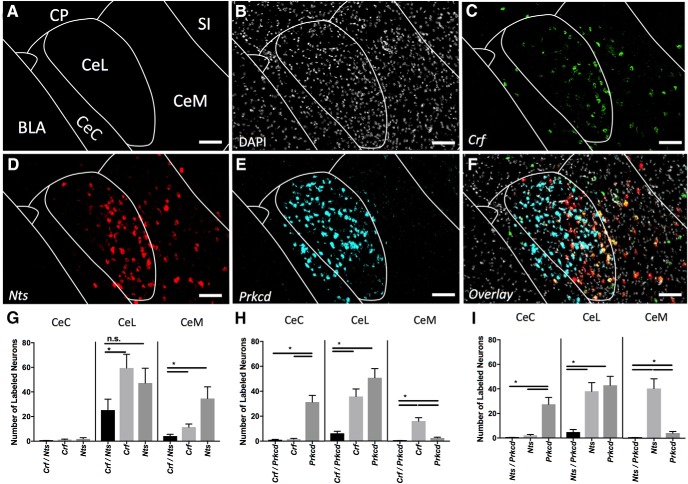
Coexpression of *Crf*, *Nts*, and *Prkcd* (A-P -1.5). ***A***, Map of area examined. ***B***, DAPI stain (gray) of area examined. ***C***, *Crf* expression (green) is found strongly in the CeL and moderately in the CeM. ***D***, *Nts* expression (red) is found strongly in the CeL and CeM. ***E***, *Prkcd* expression (cyan) is found strongly in the CeC and CeL. ***F***, Overlay of ***B–E*** reveals strong overlap in expression of *Crf* and *Nts* in CeL but not CeM. *Prkcd* does not highly coexpress in any area. Scale bar: 50 µm. ***G***, Quantification of single expressing cells and coexpressing *Crf* and *Nts* cells in CeC, CeL, and CeM. Bars represent the mean number of (co)expressing cells in each subcompartment. ***H***, Quantification of single expressing cells and coexpressing *Crf* and *Prkcd* cells in CeC, CeL, and CeM. Bars represent the mean number of (co)expressing cells in each subcompartment. ***I***, Quantification of single expressing cells and coexpressing *Nts* and *Prkcd* cells in CeC, CeL, and CeM. Bars represent the mean number of (co)expressing cells in each subcompartment. Data presented as mean ± SEM where *p < 0.05 difference between single and double-labeled populations (Mann–Whitney *U* test).

**Figure 4.**
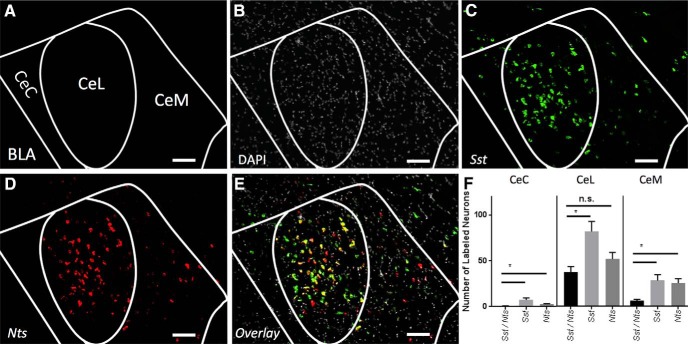
Coexpression of *Sst and Nts* (A-P -1.5). ***A***, Map of area examined ***B–E***. ***B***, DAPI stain (gray) of area examined. ***C***, *Sst* expression (green) is found strongly in the CeL and CeM. ***D***, *Nts* expression (red) is found strongly in the CeL and CeM. ***E***, Overlay of ***B–E*** reveals strong overlap in expression of *Sst* and *Nts* in CeL but not CeM. Scale bar: 50 µm. ***F***, Quantification of single expressing cells and coexpressing *Sst* and *Nts* cells in CeC, CeL, and CeM. Bars represent the mean number of (co)expressing cells in each subcompartment. Data presented as mean ± SEM where *p < 0.05 difference between single and double-labeled populations (Mann–Whitney *U* test).

**Figure 5.**
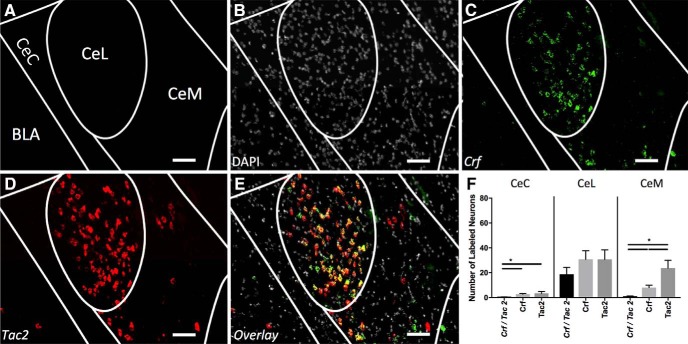
Coexpression of *Crf* and *Tac2* (A-P -1.5). ***A***, Map of area examined ***B–E***. ***B***, DAPI stain (gray) of area examined. ***C***, *Crf* expression (green) is found strongly in the CeL and CeM. ***D***, *Tac2* expression (red) is found strongly in the CeL and CeM. **E.** Overlay of B-D reveals strong overlap in expression of Crf and Tac2 in CeL but not CeM. Scale Bar indicates 50 µm. ***F***, Quantification of single expressing cells and coexpressing *Crf* and *Tac2* cells in CeC, CeL, and CeM. Bars represent the mean number of (co)expressing cells in each subcompartment. Data presented as mean ± SEM where *p < 0.05 difference between single and double-labeled populations (Mann–Whitney *U* test).

**Figure 6.**
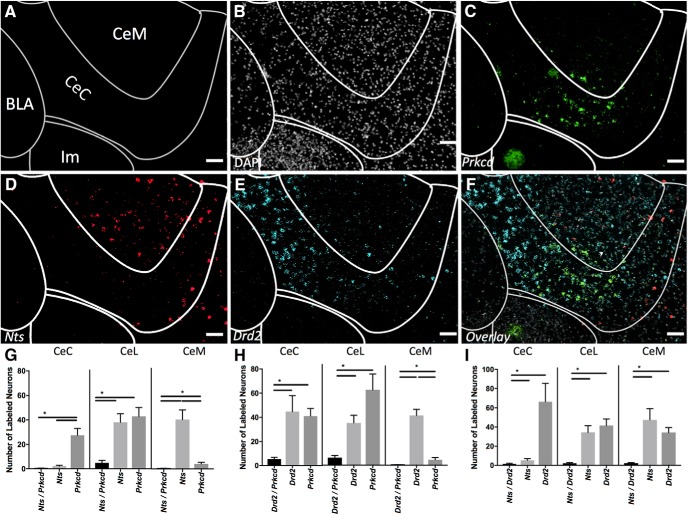
Coexpression of *Prkcd*, *Nts*, and *Drd2* (A-P -0.9). Examination of markers at anterior positions within CeA reveals differential distributions across subcompartments and reduced coexpression. ***A***, Map of area examined (A-P ∼ -0.9). ***B***, DAPI stain (gray) of area examined. ***C***, *Prkcd* expression (green) is found strongly in ventral CeC. ***D***, *Nts* expression (red) is found strongly in the CeM with limited expression in medial ventral CeC. ***E***, *Drd2* expression (cyan) is found strongly in the more dorsal elements of the CeC. ***F***, Overlay of ***B–E*** reveals limited overlap in expression of any marker examined. Scale bar: 50 µm. ***G***, Quantification of single expressing cells and coexpressing *Nts* and *Prkcd* cells in CeC, CeL, and CeM. Bars represent the mean number of (co)expressing cells in each subcompartment. ***H***, Quantification of single expressing cells and coexpressing *Drd2* and *Prkcd* cells in CeC, CeL, and CeM. Bars represent the mean number of (co)expressing cells in each subcompartment. ***I***, Quantification of single expressing cells and coexpressing *Nts* and *Drd2* cells in CeC, CeL, and CeM. Bars represent the mean number of (co)expressing cells in each subcompartment. Data presented as mean ± SEM where *p < 0.05 difference between single and double-labeled populations (Mann–Whitney *U* test).

**Figure 7.**
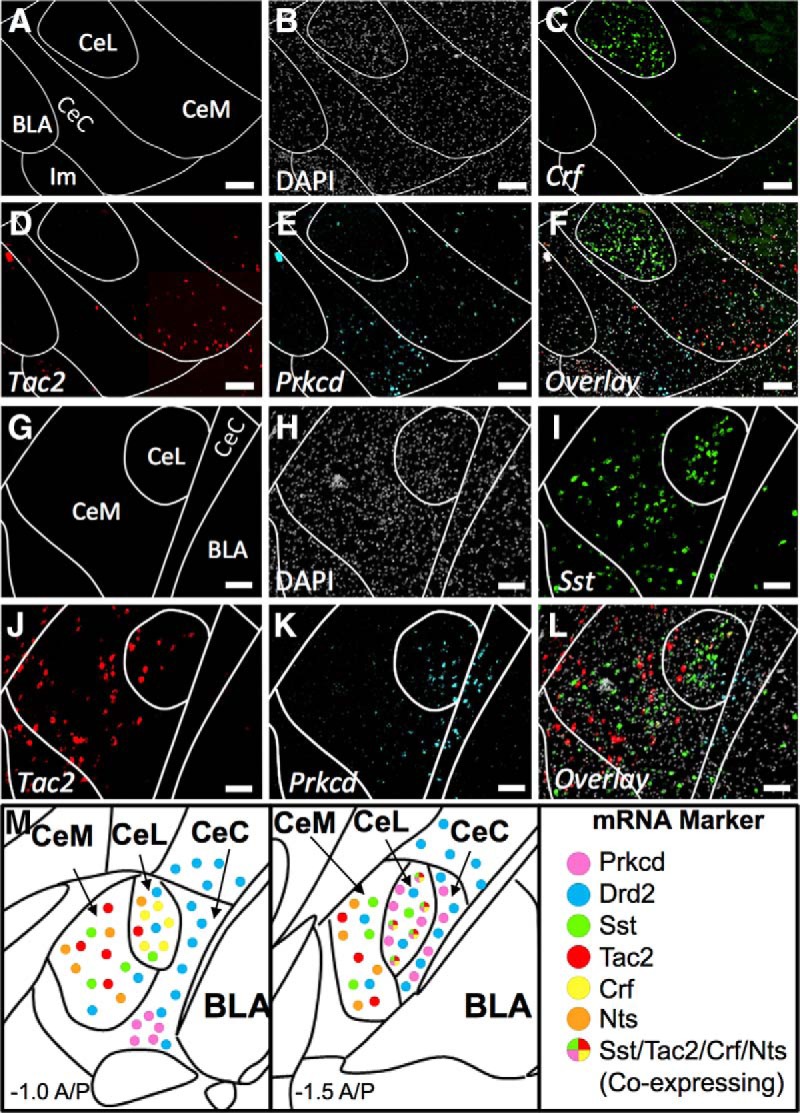
Coexpression of *Crf*, *Tac2*, and *Prkcd*; and *Sst*, *Tac2*, and *Prkcd* (A-P -0.8 and -1.22). ***A***, Map of area examined (A-P ∼-0.8). ***B***, DAPI stain (gray) of area examined. ***C***, *Crf* expression (green) is found strongly in CeL. ***D***, *Tac2* expression (red) is found in the CeM. ***E***, *Prkcd* expression (cyan) is found strongly in ventral CeC. ***F***, Overlay of ***B–E*** reveals limited overlap in expression of any marker examined. Scale bar: 200 µm. ***G***, Map of area examined (A-P ∼-1.2). ***H***, DAPI stain (gray) of area examined. ***I***, *Sst* expression (green) is found strongly in CeL and CeM. ***J***, *Tac2* expression (red) is found in the CeL and CeM. ***K***, *Prkcd* expression (cyan) is found strongly in CeL and CeC with limited expression in the CeM. ***L***, Overlay of ***H–K*** reveals limited overlap in expression of any marker examined. Scale bar: 50 µm. ***M***, Summary figure demonstrating localization and colocalization of examined CeA mRNA markers.

Colocalization among markers was examined within each CeA subcompartment. Triple-labeled images were analyzed only for colocalization between pairs of markers due to practical limitations on the number of probe combinations. Additionally, although colocalization was examined at a variety of A-P positions (A-P -0.8 to -1.8), coexpression data are presented in Figures 1–7 is collapsed across A-P -0.8 to -1.8. This may lead to an underestimation of the colocalization of some markers at certain positions (discussed below), but nonetheless provides an indication of overall colocalization between markers in the CeA. Tables 1, 2 provide quantification of expression and colocalization at both anterior (A-P -0.8 to -1.2) and posterior (A-P -1.3 to -1.8) positions.

**Table 1.** Coexpression of examined mRNAs across CeA subcompartment in anterior and posterior CeA

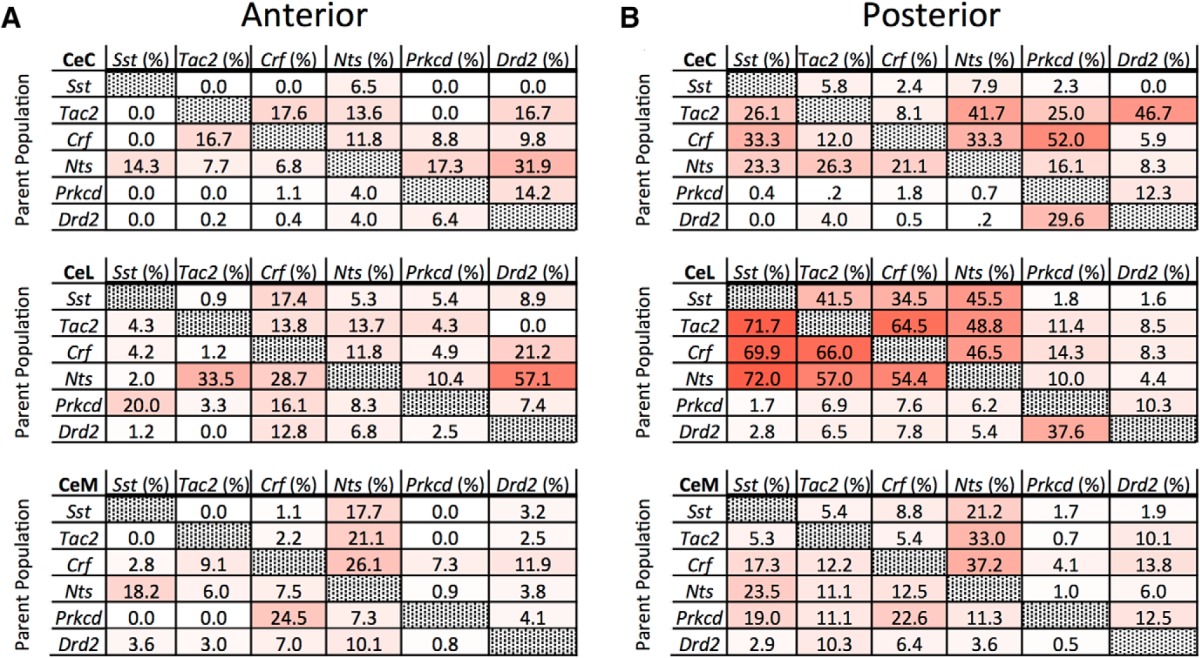

***A***, coexpression of markers of interest in anterior CeA between A-P -0.8 and -1.2. Parent population labeled on vertical column (total cells labeled). Coexpressed population labeled on horizontal column (total colabeled cells).

***B***, coexpression of markers of interest in aposterior CeA between A-P -1.3 and -1.8. Parent population labeled on vertical column (total cells labeled). Coexpressed population labeled on horizontal column (total colabeled cells).

**Table 2.** Expression and coexpression of examined mRNAs across CeA subcompartment and anterior and posterior axis

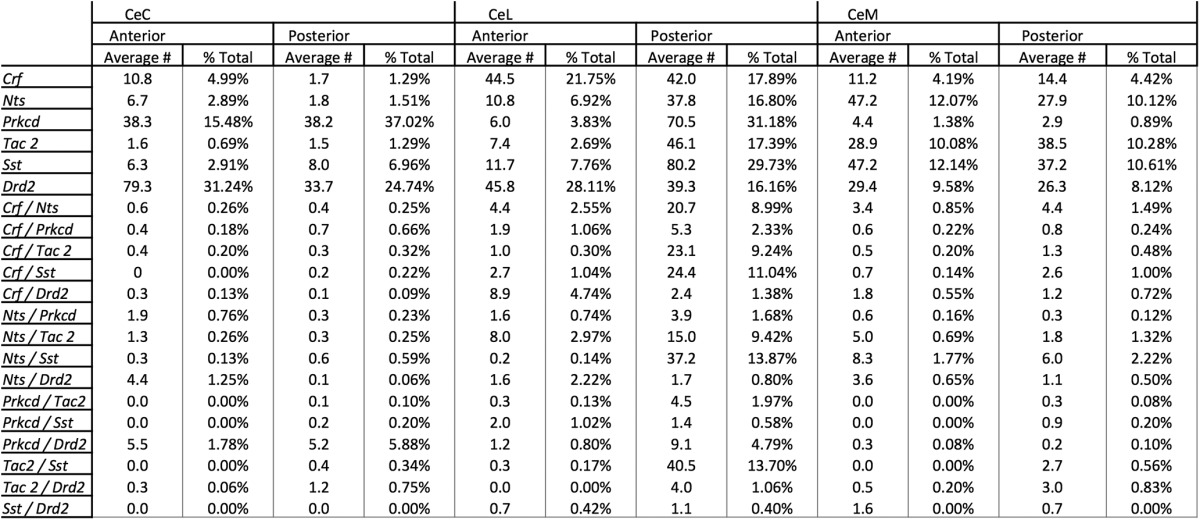

Values presented of average number of positive cells found within a subcompartment in either anterior or posterior region. Additionally, these values are presented as a percentage of total DAPI stained nuclei in each area.

### Sst/Tac 2/Prkcd

*Sst* appears to mark the largest population of CeL cells (Figs. 1*C*, 2*C* ). This population overlaps to a great extent with *Tac2* (Fig. 2*D*) within the CeL, but not the CeC or CeM (Fig. 2*F*). Quantification of coexpression reveals that the total *Sst* labeled population is significantly larger than the coexpressing *Sst/Tac2* population in all subcompartments; however, within the CeL the *Tac2* population is not significantly different from the coexpressing *Sst/Tac2* population (Fig. 2*G*). These data suggest that the larger *Sst* population may entirely contain the *Tac2* population at this A-P range. *Prkcd* exhibits a typical dense CeC and CeL expression (Fig. 2*E*). Within the CeL total *Sst, Tac2* and *Prkcd* populations are larger than coexpressing populations, suggesting these RNAs mark separate populations (Fig. 2*H*,*I*). Within CeC and CeM total populations are significantly larger than coexpressing populations except in cases where total population is very small.

### Crf/Nts/Prkcd

*Crf* labels a large population of CeL cells with somewhat sparser labeling in the CeM (Fig. 3*C*). *Nts* marks a large population within the CeL and a moderate population within the CeM (Fig. 3*D*). Quantification of coexpression reveals the total *Crf* population is significantly larger than the coexpressing *Crf/Nts* population in the CeL and CeM; however, within the CeL the *Nts* population is not significantly different from the coexpressing Crf*/Nts* population (Fig. 3*G*). This suggests that within the CeL, the *Nts* population may be contained within the *Crf* population, while within the CeM, these populations are distinct. *Prkcd* demonstrates a similar expression pattern to that seen in Figure 2 (Fig. 3*E*). The *Prkcd* population is separately expressed from the *Crf* and *Nts* populations in all areas where an appreciable number of marked cells are found (Fig. 3*H*,*I*).

### Nts/Sst

Examination of coexpression of *Nts* and *Sst* reveals similar patterns. Within the CeL the *Nts* population appears to be contained within the *Sst* population (Fig. 4*A–F*). While within the CeM these mRNAs mark distinct populations (Fig. 4*F*).

### Crf/Tac 2

Likewise, when *Crf* and *Tac2* are examined for coexpression, neither of the total labeled populations is significantly larger than the colabeled *Crf/Tac2* populations (Fig. 5*C–F*). However, within the CeM these mRNAs mark separate populations (Fig. 5*C–F*).

These results suggest a hierarchical organization within the CeL wherein *Sst > Crf ∼Tac2 >Nts*. This is in contrast to the CeM where all total labeled populations are found to be significantly different from their colabeling with any other marker.

Examination of coexpression at a variety of A-P positions reveals that the zone of highest coexpression between *Sst*, *Tac2*, *Nts*, and *Crf* is constrained to A-P -1.3 to -1.8. Examination of more anterior positions (A-P- 0.8 to -1.2) demonstrates that these populations are coexpressed at lower rates and found in different subcompartments in the anterior CeA.

### Prkcd/Nts/Drd2

At anterior positions (A-P ∼-0.9) *Drd2* labels a large population of CeC cells while *Prkcd* cells are found in a cluster in the ventral aspect of the CeC (Fig. 6*C*,*E*). At this A-P position *Nts* is found primarily within the CeM (Fig. 6*D*). These populations largely do not overlap (Fig. 6*F–I*).

### Crf/Tac 2/Prkcd/Sst

At a similar A-P position (∼-0.8) *Crf* densely labels the CeL (Fig. 7*C*). Very little *Tac2* staining is found within the CeL; however, labeled *Tac2* cells are found in the CeM and the dorsal aspect of the main intercalated mass (Im) located ventrally to the BLA (Fig. 7*D*). *Prkcd* is found in the ventral CeC (Fig. 7*E*). These populations largely do not overlap (Fig. 7*F*).

Slightly more posteriorly (A-P ∼-1.2), the densely labeled CeL seen in more posterior sections begins to appear (Fig. 7*G–L*). *Sst* densely labels the CeL and more sparsely the CeM (Fig. 7*I*). *Prkcd* begins to form the typical CeC and CeL expression pattern (Fig. 7*K*); however, *Tac2* does not densely label the CeL at this position and markers continue to be coexpressed at low levels (Fig. 7*J–L*). These results highlight that the zone of dense coexpression in CeL is constrained to more posterior aspects of the CeA.

The overall percentages of coexpression for all mRNA pairs examined at anterior and posterior positions is presented in Table 1. Table 2 contains quantification of total numbers of labeled and colabeled cells as well as labeled cells as a percentage of total DAPI-labeled cells. Descriptive summary of localization and colocalization of examined CeA mRNA markers is found in Figure 7*M*.

## Discussion

The central amygdala plays a pivotal role in the control of a wide range of behaviors, including those reflecting fear and anxiety (Maren and Fanselow, 1996; Johansen et al., 2011; Pare and Duvarci, 2012; Fadok et al., 2017; Lezak et al., 2017). As such, the connectivity, cytoarchitecture and expression profiles of cells in the various subdivisions of this nucleus have been widely studied, especially in rats (McDonald, 1982, 1984; Jolkkonen and Pitkänen, 1998; McDonald, 1998; Cassell et al., 1999). To date, a number of molecularly identified populations have been described as playing distinct roles in the control of behavior. Additionally, the distributions of these populations have been mapped using immunohistochemistry or *in situ* hybridization (Cassell et al., 1986; Cassell et al., 1999; Andero et al., 2014; Andero et al., 2016). However, minimal data are available on the extent to which these population markers overlap, especially in mice, leading to ambiguity in the specificity of identified and manipulated populations. The present data provide a novel and important advance by examining the coexpression of *Prkcd*, *Sst*, *Nts*, *Tac2*, *Crf*, and *Drd2* in the CeA of mice. Additionally, these data begin to address the critical need for parsimonious description of both molecular and locational identity of neuron populations examined in the CeA.

Our methods for identifying cells expressing an mRNA involved a binary system so that all cells reaching minimum cutoff (five fluorescent puncta within twice the nuclear diameter) were considered to be expressing. Thus, the representation of low expressing transcripts may be under sampled in exchange for increased confidence. This is most relevant for *Drd2*, which has the greatest apparent range in expression strength. All animals were the same age and brains were obtained under the same conditions at the same time. This approach was used to characterize the baseline identity of cells; however, it ignores a wealth of data concerning levels of expression at the time of sacrifice and dynamic (e.g., circadian or following behavior) changes in expression level. One clear example is that both *Tac2* and *Sst* are clearly expressed at different levels in different populations. Cells appear to express *Tac2* and *Sst* at both high (bright) and moderate/low (dimmer) levels within the same subnucleus. Future studies examining static differences and dynamic changes in mRNA expression level may yield important information regarding the functional roles of these mRNAs.

Our data confirm in mice previous immunohistochemical analyses conducted in rats by demonstrating that within the CeL there is a high degree of overlap between *Sst*, *Nts*, *Tac2*, and *Crf*. Remarkably, this overlap is observed only within a constrained posterior section of the CeL between A-P -1.4 and -1.8. Examination of these populations across the A-P axis suggests that *Crf* most consistently marks a CeL population while *Sst*, *Nts*, and *Tac2* most consistently label cells in the anterior CeM before densely marking the CeL at posterior positions. Within the posterior CeL, these populations are highly overlapping. *Sst*-expressing cells represent the largest population containing the majority of cells expressing *Nts*, *Tac2*, and *Crf*. This is in contrast to the CeM where these populations are consistently nonoverlapping.

An important consideration for the examination of these populations across the A-P axis is the inconsistency across currently available mouse brain atlases. For example, at anterior positions the Allen Brain atlas identifies the location of the dense *Crf* population as the CeL while the Paxinos and Franklin (2013) atlas identifies this region as the interstitial nucleus of the posterior limb of the anterior commissure (IPAC). While these may be semantic differences, the consistency of nucleus identification has important implication for the quantification of coexpression. Our decision to adhere more closely to the Allen Brain Atlas Reference Atlas may have led to an underestimation of the extent of coexpression of examined markers within the CeL.

*Drd2* appears to consistently mark a large CeC and CeL population that is contiguous with the Ast. This is in contrast to *Prkcd*, which at anterior positions marks a very ventral population of CeC cells before moving more dorsally to mark a very constrained population of CeC and CeL cells at posterior positions. Consistent with previously published work, neither the *Prkcd* nor the *Drd2* populations is highly overlapping with any others examined. This finding validates the identification of these populations as potentially markers for functionally distinct sub populations (Kim et al., 2017).

Literature identifying functionally distinct CeA populations has been inconsistent in identifying precisely the CeA subcompartment where neurons of interest reside. Such a specific delineation is especially critical in the case of *Sst*, *Nts*, *Tac2*, and *Crf*, where the identification of these populations within the CeL may be redundant to previous work. Conversely, lack of coexpression in the CeM highlighted by the present findings may indicate a more specialized role for these cells. Future studies using intersectional approaches may yield clear and parsimonious descriptions of the distinct functional roles of single expressing and coexpressing populations in the CeL and CeM (Dymecki et al., 2010; Hirsch et al., 2013; Jensen and Dymecki, 2014; Okaty et al., 2015). Additionally, unlike in the CeL where examined populations label a majority of total cells, in the CeM, examined populations make up less than half of total cells indicating many additional populations that remain to be described.

These results represent a starting point in a more comprehensive characterization of the many possible markers for CeA subpopulations. The receptors of the protein products of several of the mRNAs examined may be promising markers for specific subpopulations (*Crfr1*, *Crfr2*, *Tacr2*, *Sstr1-5*). Additionally, further research into the coexpression of various neuropeptides and other identified markers such as pituitary adenylate cyclase-activating polypeptide (PACAP), vasoactive intestinal peptide, cholecystokinin, neuropeptide Y, dynorphin, enkephalin, and substance P (all of which have also been shown to also play important roles in fear and anxiety behaviors) will, in the future, be necessary to identify the extent to which additional populations colocalize within the CeA. These types of analyses provide a more thorough understanding of the molecular basis of amygdala function and may facilitate the development of innovative approaches, such as those target-specific cell types by exploiting their unique patterns of receptor expression, to treat fear and anxiety-related disorders.

## Synthesis

Reviewing Editor: Carmen Sandi, Swiss Federal Institute of Technology Decisions are customarily a result of the Reviewing Editor and the peer reviewers coming together and discussing their recommendations until a consensus is reached. When revisions are invited, a fact-based synthesis statement explaining their decision and outlining what is needed to prepare a revision will be listed below. Note: If this manuscript was transferred from JNeurosci and a decision was made to accept the manuscript without peer review, a brief statement to this effect will instead be what is listed below.

This manuscript presents a molecular identification and localization of neurons in different compartments of the mouse central amygdala (CeA), including capsular (CeC), lateral (CeL), and medial (CeM) parts. By performing three-color in situ hybridization, this article shows expression for somatostatin (Sst), neurotensin (Nts), corticotropin releasing factor (Crf), tachykinin 2 (Tac2), protein kinase c delta (Prkcd), and dopamine receptor 2 (Drd2) mRNA. The study reveals specific patterns of expression and co-localization of these markers, which in some regions show a great overlap. Therefore, this work elegantly illustrates neuronal identity in a brain region highly studied and targeted in studies that aim at revealing cell-specific functional identity, particularly in the field of fear circuits. Accordingly, the reported data advances knowledge on cell types and circuits in the CeA and can be instrumental in guiding future work targeting cells containing the examined molecular markers.

## References

[B1] Abraham AD, Neve KA, Lattal KM (2014) Dopamine and extinction: a convergence of theory with fear and reward circuitry. Neurobiol Learn Mem 108:65–77. 24269353 10.1016/j.nlm.2013.11.007 PMC3927738

[B2] Andero R, Dias BG, Ressler KJ (2014) A role for Tac2, NkB, and Nk3 receptor in normal and dysregulated fear memory consolidation. Neuron 83:444–454. 24976214 10.1016/j.neuron.2014.05.028 PMC4103970

[B3] Andero R, Daniel S, Guo JD, Bruner RC, Seth S, Marvar PJ, Rainnie D, Ressler KJ (2016) Amygdala-dependent molecular mechanisms of the Tac2 pathway in fear learning. Neuropsychopharmacology 41:2714–2722. 27238620 10.1038/npp.2016.77 PMC5026739

[B4] Bourgeais L, Gauriau C, Bernard JF (2001) Projections from the nociceptive area of the central nucleus of the amygdala to the forebrain: a PHA-L study in the rat. Eur J Neurosci 14:229–255. 11553276 10.1046/j.0953-816x.2001.01640.x

[B5] Cai H, Haubensak W, Anthony TE, Anderson DJ (2014) Central amygdala PKC-δ(+) neurons mediate the influence of multiple anorexigenic signals. Nat Neurosci 17:1240–1248. 10.1038/nn.3767 25064852 PMC4146747

[B6] Cassell MD, Gray TS, Kiss JZ (1986) Neuronal architecture in the rat central nucleus of the amygdala: a cytological, hodological, and immunocytochemical study. J Comp Neur 246:478–499. 10.1002/cne.902460406 2422231

[B7] Cassell MD, Freedman LJ, Shi C (1999) The intrinsic organization of the central extended amygdala. Ann NY Acad Sci 877:217–241. 10415652 10.1111/j.1749-6632.1999.tb09270.x

[B8] Ciocchi S, Herry C, Grenier F, Wolff SB, Letzkus JJ, Vlachos I, Ehrlich I, Sprengel R, Deisseroth K, Stadler MB, Müller C, Lüthi A (2010) Encoding of conditioned fear in central amygdala inhibitory circuits. Nature 468:277–282. 10.1038/nature09559 21068837

[B9] de la Mora MP, Gallegos-Cari A, Arizmendi-García Y, Marcellino D, Fuxe K (2010) Role of dopamine receptor mechanisms in the amygdaloid modulation of fear and anxiety: structural and functional analysis. Prog Neurobiol 90:198–216. 10.1016/j.pneurobio.2009.10.010 19853006

[B10] Dymecki SM, Ray RS, Kim JC (2010) Mapping cell fate and function using recombinase-based intersectional strategies. Methods Enzymol 477:183–213. 10.1016/S0076-6879(10)77011-7 20699143

[B11] Ehrlich I, Humeau Y, Grenier F, Ciocchi S, Herry C, Lüthi A (2009) Amygdala inhibitory circuits and the control of fear memory. Neuron 62:757–771. 10.1016/j.neuron.2009.05.026 19555645

[B12] Fadok JP, Krabbe S, Markovic M, Courtin J, Xu C, Massi L, Botta P, Bylund K, Müller C, Kovacevic A, Tovote P, Lüthi A (2017) A competitive inhibitory circuit for selection of active and passive fear responses. Nature 542:96–100. 10.1038/nature21047 28117439

[B13] Gafford GM, Ressler KJ (2015) GABA and NMDA receptors in CRF neurons have opposing effects in fear acquisition and anxiety in central amygdala vs. bed nucleus of the stria terminalis. Horm Behav 76:136–142. 10.1016/j.yhbeh.2015.04.001 25888455 PMC4844457

[B14] Haubensak W, Kunwar PS, Cai H, Ciocchi S, Wall NR, Ponnusamy R, Biag J, Dong HW, Deisseroth K, Callaway EM, Fanselow MS, Lüthi A, Anderson DJ (2010) Genetic dissection of an amygdala microcircuit that gates conditioned fear. Nature 468:270–276. 10.1038/nature09553 21068836 PMC3597095

[B15] Herry C, Ciocchi S, Senn V, Demmou L, Müller C, Lüthi A (2008) Switching on and off fear by distinct neuronal circuits. Nature 454:600–606. 10.1038/nature07166 18615015

[B16] Hirsch MR, d' Autréaux F, Dymecki SM, Brunet JF, Goridis C (2013) A Phox2b::FLPo transgenic mouse line suitable for intersectional genetics. Genesis 51:506–514. 10.1002/dvg.22393 23592597 PMC4036463

[B17] Jensen P, Dymecki SM (2014) Essentials of recombinase-based genetic fate mapping in mice. Methods Mol Biol 1092:437–454. 10.1007/978-1-60327-292-6_26 24318835 PMC4035813

[B18] Johansen JP, Cain CK, Ostroff LE, LeDoux JE (2011) Molecular mechanisms of fear learning and memory. Cell 147:509–524. 10.1016/j.cell.2011.10.009 22036561 PMC3215943

[B19] Jolkkonen E, Pitkänen A (1998) Intrinsic connections of the rat amygdaloid complex: projections originating in the central nucleus. J Comp Neur 395:53–72. 9590546 10.1002/(sici)1096-9861(19980525)395:1<53::aid-cne5>3.0.co;2-g

[B20] Kim J, Zhang X, Muralidhar S, LeBlanc SA, Tonegawa S (2017) Basolateral to central amygdala neural circuits for appetitive behaviors. Neuron 93:1464–1479.e5. 10.1016/j.neuron.2017.02.034 28334609 PMC5480398

[B21] Kwon OB, Lee JH, Kim HJ, Lee S, Lee S, Jeong MJ, Kim SJ, Jo HJ, Ko B, Chang S, Park SK, Choi YB, Bailey CH, Kandel ER, Kim JH (2015) Dopamine regulation of amygdala inhibitory circuits for expression of learned fear. Neuron 88:378–389. 10.1016/j.neuron.2015.09.001 26412489

[B22] Lein ES, Hawrylycz MJ, Ao N, Ayres M, Bensinger A, Bernard A, Boe AF, Boguski MS, Brockway KS, Byrnes EJ, Chen L, Chen L, Chen TM, Chin MC, Chong J, Crook BE, Czaplinska A, Dang CN, Datta S, Dee NR, et al. (2007) Genome-wide atlas of gene expression in the adult mouse brain. Nature 445:168–176. 10.1038/nature05453 17151600

[B23] Letzkus JJ, Wolff SB, Lüthi A (2015) Disinhibition, a circuit mechanism for associative learning and memory. Neuron 88:264–276. 10.1016/j.neuron.2015.09.024 26494276

[B24] Lezak KR, Missig G, Carlezon WA Jr (2017) Behavioral methods to study anxiety in rodents. Dialogues Clin Neurosci 19:181–191. 28867942 10.31887/DCNS.2017.19.2/wcarlezon PMC5573562

[B25] Li H, Penzo MA, Taniguchi H, Kopec CD, Huang ZJ, Li B (2013a) Experience-dependent modification of a central amygdala fear circuit. Nat Neurosci 16:332–339. 10.1038/nn.3322 23354330 PMC3581751

[B26] Li H, Penzo MA, Taniguchi H, Kopec CD, Huang ZJ, Li B (2013b) Experience-dependent modification of a central amygdala fear circuit. Nat Neurosci 16:332–339. 10.1038/nn.3322 23354330 PMC3581751

[B27] Maren S, Fanselow MS (1996) The amygdala and fear conditioning: has the nut been cracked? Neuron 16:237–240. 8789938 10.1016/s0896-6273(00)80041-0

[B28] McCullough KM, Morrison FG, Ressler KJ (2016) Bridging the Gap: towards a cell-type specific understanding of neural circuits underlying fear behaviors. Neurobiol Learn Mem 135:27–39. 10.1016/j.nlm.2016.07.025 27470092 PMC5123437

[B29] McDonald AJ (1982) Cytoarchitecture of the central amygdaloid nucleus of the rat. J Comp Neur 208:401–418. 10.1002/cne.902080409 7119168

[B30] McDonald AJ (1984) Neuronal organization of the lateral and basolateral amygdaloid nuclei in the rat. J Comp Neur 222:589–606. 10.1002/cne.902220410 6199387

[B31] McDonald AJ (1998) Cortical pathways to the mammalian amygdala. Prog Neurobiol 55:257–332. 9643556 10.1016/s0301-0082(98)00003-3

[B32] McDonald AJ (2003) Is there an amygdala and how far does it extend? An anatomical perspective. Ann NY Acad Sci 985:1–21. 12724144 10.1111/j.1749-6632.2003.tb07067.x

[B33] Merali Z, McIntosh J, Kent P, Michaud D, Anisman H (1998) Aversive and appetitive events evoke the release of corticotropin-releasing hormone and bombesin-like peptides at the central nucleus of the amygdala. J Neurosci 18:4758–4766. 9614249 10.1523/JNEUROSCI.18-12-04758.1998 PMC6792703

[B34] Okaty BW, Freret ME, Rood BD, Brust RD, Hennessy ML, deBairos D, Kim JC, Cook MN, Dymecki SM (2015) Multi-scale molecular deconstruction of the serotonin neuron system. Neuron 88:774–791. 10.1016/j.neuron.2015.10.007 26549332 PMC4809055

[B35] Pare D, Duvarci S (2012) Amygdala microcircuits mediating fear expression and extinction. Curr Opin Neurobiol 22:717–723. 10.1016/j.conb.2012.02.014 22424846 PMC3380167

[B43] Paxinos G, Franklin K (2013) Paxinos and Franklin's the mouse brain in stereotaxic coordinates Elsevier/Academic Press Boston, MA.

[B36] Penzo MA, Robert V, Li B (2014) Fear conditioning potentiates synaptic transmission onto long-range projection neurons in the lateral subdivision of central amygdala. J Neurosci 34:2432–2437. 10.1523/JNEUROSCI.4166-13.2014 24523533 PMC3921418

[B37] Perez de la Mora M, Gallegos-Cari A, Crespo-Ramirez M, Marcellino D, Hansson AC, Fuxe K (2012) Distribution of dopamine D(2)-like receptors in the rat amygdala and their role in the modulation of unconditioned fear and anxiety. Neuroscience 201:252–266. 10.1016/j.neuroscience.2011.10.045 22100273

[B38] Petrovich G, Swanson L (1997) Projections from the lateral part of the central amygdalar nucleus to the postulated fear conditioning circuit. Brain Res 763:247–254. 9296566 10.1016/s0006-8993(96)01361-3

[B39] Shilling PD, Feifel D (2008) The neurotensin-1 receptor agonist PD149163 blocks fear-potentiated startle. Pharmacol Biochem Behav 90:748–752. 10.1016/j.pbb.2008.05.025 18577396 PMC4215568

[B40] Thompson BL, Erickson K, Schulkin J, Rosen JB (2004) Corticosterone facilitates retention of contextually conditioned fear and increases CRH mRNA expression in the amygdala. Behav Brain Res 149:209–215. 15129783 10.1016/s0166-4328(03)00216-x

[B41] Yamauchi R, Wada E, Kamichi S, Yamada D, Maeno H, Delawary M, Nakazawa T, Yamamoto T, Wada K (2007) Neurotensin type 2 receptor is involved in fear memory in mice. J Neurochem 102:1669–1676. 10.1111/j.1471-4159.2007.04805.x 17697051

[B42] Yu K, Garcia da Silva P, Albeanu DF, Li B (2016) Central amygdala somatostatin neurons gate passive and active defensive behaviors. J Neurosci 36:6488–6496. 10.1523/JNEUROSCI.4419-15.2016 27307236 PMC5015784

